# Artificial intelligence assisted operative anatomy recognition in endoscopic pituitary surgery

**DOI:** 10.1038/s41746-024-01273-8

**Published:** 2024-11-09

**Authors:** Danyal Z. Khan, Alexandra Valetopoulou, Adrito Das, John G. Hanrahan, Simon C. Williams, Sophia Bano, Anouk Borg, Neil L. Dorward, Santiago Barbarisi, Lucy Culshaw, Karen Kerr, Imanol Luengo, Danail Stoyanov, Hani J. Marcus

**Affiliations:** 1 Department of Neurosurgery, National Hospital for Neurology and Neurosurgery, London, UK; 2 Hawkes Centre, Department of Computer Science, University College London, London, UK; 3 Digital Surgery Ltd, Medtronic, London, UK

**Keywords:** Brain, Computational science

## Abstract

Pituitary tumours are surrounded by critical neurovascular structures and identification of these intra-operatively can be challenging. We have previously developed an AI model capable of sellar anatomy segmentation. This study aims to apply this model, and explore the impact of AI-assistance on clinician anatomy recognition. Participants were tasked with labelling the sella on six images, initially without assistance, then augmented by AI. Mean DICE scores and the proportion of annotations encompassing the centroid of the sella were calculated. Six medical students, six junior trainees, six intermediate trainees and six experts were recruited. There was an overall improvement in sella recognition from a DICE of score 70.7% without AI assistance to 77.5% with AI assistance (+6.7; *p* < 0.001). Medical students used and benefitted from AI assistance the most, improving from a DICE score of 66.2% to 78.9% (+12.8; *p* = 0.02). This technology has the potential to augment surgical education and eventually be used as an intra-operative decision support tool.

## Introduction

Pituitary tumours are amongst the most common intracranial tumours, and are located in an anatomically dense region of the body. These tumours often compress, distort or encase surrounding critical neurovascular structures, such as the optic nerves and internal carotid arteries, often manifesting in symptoms such as visual deficits^1^. During surgery, which is most commonly performed via an endonasal transsphenoidal approach, identification and protection of these structures is a core operative step and paramount to preventing harm^2,3^.

Identification of these structures intra-operatively can be facilitated by anatomical landmarks, most notably, impressions of these anatomical structures on the base of the sphenoid bone^4^. However, these bony landmarks are variable (e.g. variations in sphenoid sinus aeration and septations), and are often distorted by tumours, particularly giant adenomas, making intraoperative anatomy orientation challenging. Similarly, previous skull base surgery, radiotherapy or concomitant sinonasal pathology may distort local structures and add to this challenge.

Endonasal transsphenoidal surgery has seen numerous technological advancements in recent years, many of which have aided intraoperative navigation and protection of critical surrounding tissue^5^. Firstly, the use of an endoscopic approach improves the width of vision at the end of a long endonasal corridor. However, the majority of endoscopes are 2D, and therefore lack depth perception. Similarly, the application of image-guided surgical systems, or “neuronavigation”, assists in anatomical orientation based on pre-operative imaging but does not account for intra-operative tissue shifts^6^. Furthermore, the application of micro-Doppler (to identify arterial/venous structures) and neurophysiological monitoring (e.g. optic nerve monitoring) may be used in selected cases, with the advantage of being “real-time” but requiring extra equipment and expertise^7^.

More recently, the use of computer vision and machine learning (particularly deep learning) is being increasingly used to analyse operative video data and recognise surgical steps, anatomy and instruments^2,8–10^. This technology has the potential to be both real-time and integrate seamlessly into the existing operative workflow^5^. Automated recognition of nasal structures has previously been explored but has not yet been achieved for the critical sellar and para-sellar structures^11^. We have previously developed a pre-clinical AI (Artificial Intelligence) model capable of accurate sellar anatomy recognition^8,9^. In this pre-clinical IDEAL (Idea, Development, Exploration, Assessment, Long-term study) Stage 0 study, we sought to apply this AI model, and evaluate its performance as an AI assistant for clinicians in anatomical structure recognition during endoscopic pituitary adenoma resection.

## Results

### General characteristics

Twenty-four participants took part in this pre-clinical comparative study, including six medical students, six junior neurosurgical trainees, six intermediate trainees and six experts (17 male, 7 female) from a single tertiary neurosurgical centre.

### Comparative evaluation of human-only vs AI-assisted anatomy recognition

**Fig. 1:**
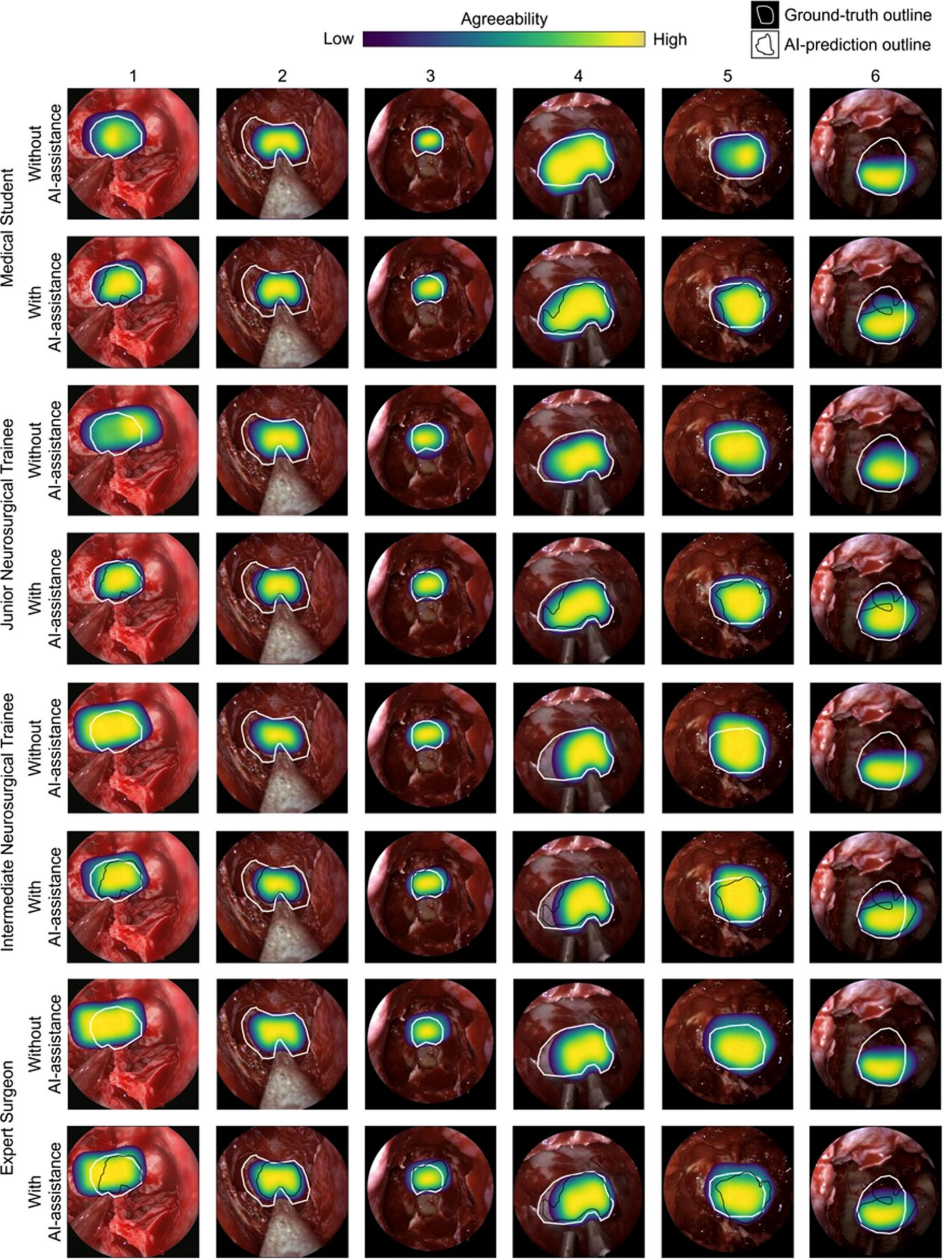
Heat map of participant sella annotations on each of the six images before and after AI assistance, per subgroup. Areas of high agreeability between annotations are shown in yellow and indicate significant overlap of sella annotations amongst participants of a subgroup. For context, the outlines of the ground truth (white border) and AI suggested (black border) sella labels are shown. Comparison of heat maps pre and post AI assistance for each subgroup summarises the spatial changes made after AI assistance.

When considering the centroid of the sella only (rather than recognising the whole sella), most participants encompassed this within their annotations without AI assistance (19/24 participants, 79.2%), which improved with statistical significance to 24/24 (100%) with AI assistance (*p* = 0.025) (Table 1, Fig. 1). Medical students and junior trainees showed the most improvement, with 4/6 participants in each group recognising the sella centroid without AI assistance, and 6/6 participants in each group recognising it with AI assistance (+33.3%; *p* = 0.157). Intermediate trainees improved less dramatically (5/6 participants pre AI assistance, 6/6 participants post AI assistance; *p* = 0.317). All experts recognised the sella centroid without and with AI assistance.

**Table 1:** Proportion of annotations including the sella centroid

	Proportion of annotations including the sella centroid, without AI assistance (%)	Proportion of annotations including the sella centroid, with AI assistance (%)	Difference (%)	*p* value^a^
Overall	136/144 (94.4%)	144/144 (100%)	+5.6	0.007
Medical students	31/36 (86.1%)	36/36 (100%)	+13.9	0.054
Junior trainees	34/36 (94.4%)	36/36 (100%)	+5.6	0.493
Intermediate trainees	35/36 (97.2%)	36/36 (100%)	+2.8	1.0
Experts	36/36 (100%)	36/36 (100%)	0	–

^a^*p* values calculated using the Fisher’s Exact Test.

Regarding whole sella recognition, without AI assistance, experts had the highest DICE scores (73.4%, SD 7.8), followed by junior trainees (72.1%, SD 13.6), intermediate trainees (71.6%, SD 18.0) and medical students (66.2%, SD 13.0) (Table 2). Overall, there was an improvement in sella recognition across all subgroups from a DICE of score 70.7% without AI assistance to 77.5% with AI assistance (+6.7; *p* < 0.001). Medical students benefitted the most from AI assistance, from a DICE score of 66.2% without AI assistance to 78.9% with AI assistance (+12.8; *p* = 0.02). Junior trainees had the second highest improvement from a DICE score of 72.1% without AI assistance to 80.1% with AI assistance (+8.1; *p* < 0.001). This was followed by intermediate trainees from DICE 71.6% without AI assistance to 76.3% with AI assistance (+4.8; *p* = 0.001). Experts experienced the least improvement from a DICE score of 73.4% without AI assistance to 74.5% with AI assistance (+1.2; *p* = 0.032) (Table 2).

**Table 2:** Mean DICE scores of whole sella recognition without and with AI assistance

	Mean DICE^a^ score without AI assistance	Mean DICE score with AI assistance	Difference in mean DICE	*p* value^b^
Overall	70.7% (SD 18.0)	77.5% (SD 9.0)	+6.7	< 0.001
Medical students	66.2% (SD 13.0)	78.9% (SD 6.3)	+12.8	0.020
Junior trainees	72.1% (SD 13.6)	80.1% (SD 9.4)	+8.1	< 0.001
Intermediate trainees	71.6% (SD 11.2)	76.3% (SD 8.2)	+4.8	0.001
Experts	73.4% (SD 7.8)	74.5% (SD 8.1)	+1.2	0.032

*SD* standard deviation.

^a^DICE score of 100 indicates maximum overlap between annotated images and the ground truth annotation.

^b^*p* values calculated using the Wilcoxon Signed-Rank Test, pairing for each participant-image combination.

Overall, 76/144 (53.8%) sella annotations were changed following AI assistance. Medical students and junior trainees were more likely to change their annotation following AI assistance – 25/36 (69.4%) annotations changed compared to intermediate trainees 12/36 (33.3%) annotations changed and experts 14/36 (38.9%) annotations changed. Examining both the positive and negative impact of these sella annotation changes reveals that students were again most likely to improve DICE performance with AI (66.7% of images) but also most likely to reduce performance with AI (22.2% of images), with no change in performance in 11.1% of images. Conversely, experts were least likely to change performance with AI assistance (47.2% of images), and whilst their performance was only improved on 36.1% of images, they had the lowest reduction of performance post-AI (16.7% of images) – tying with senior trainees. These senior trainees also reduced performance with AI assistance in 16.7% of images but improved (38.9%) or had no change in performance (44.4%) for the majority of images. Intermediate trainees post AI assistance improved in 61.1% of images, did not change in 19.4% of images and worsened in 19.4% of images.

Both false positive and false negative errors were reduced overall with AI assistance, with false positives reducing to a higher magnitude than false negatives for all groups except for students (Table 3). Figure 1 depicts the spatial aspect of the impact of AI assistance on false positive and false negative errors across participant subgroups.

**Table 3:** Summary of false positive and false negative error analysis in whole sella recognition, pre and post AI assistance

	Mean false positive error (%)			Mean false negative error (%)		
	Pre-AI assistance	Post-AI assistance	Change	Pre-AI assistance	Post-AI assistance	Change
**Overall**	24.6 (SD 14.2)	19.8 (SD 11.7)	**−4.8 (SD −2.5)**	20.7 (SD 12.9)	17 (SD 10.7)	**−3.7 (SD −2.2)**
**Medical students**	22.4 (SD 13.1)	17.6 (SD 10)	**−4.8 (SD −3.2)**	28.2 (SD 21.9)	17.3 (SD 10.7)	**−10.9 (SD −11.2)**
**Junior trainees**	24.9 (SD 14.4)	16.2 (SD 11.4)	**−8.7 (SD −3)**	18.8 (SD 9.9)	17 (SD 13.2)	**−1.8 (SD 3.3)**
**Intermediate trainees**	26.6 (SD 13.6)	21.9 (SD 14.6)	**−4.7 (SD 0.9)**	17.7 (SD 11)	16.4 (SD 12.5)	**−1.3 (SD 1.5)**
**Experts**	24 (SD 15.5)	23.2 (SD 18.6)	**−0.8 (SD 3.1)**	18.1 (SD 8.9)	17.4 (SD 16.5)	**−0.7 (SD 7.7)**

*AI* artificial intelligence; *SD* standard deviation.

Values are given as a percentage of the total combined area of the overlap between the ground truth and prediction segmentations.

## Discussion

We present the first comparative study of human-only vs AI assisted intra-operative anatomy recognition in endoscopic pituitary adenoma surgery. AI assistance was found to improve recognition of the sella (safe entry zone) across different levels of expertise. Similarly, AI assistance appeared to reduce the incidence of false positive and false negative errors, supporting its potential for improving surgical safety. False positive errors were reduced to a greater magnitude, which is reassuring as clinically these errors are more dangerous, for example, leading to inadvertent damage of a critical non-sella structure (e.g. carotid artery) through incorrectly recognising it as part of the sella safe entry zone.

Surgical safety relies on the recognition and clear delineation of intra-operative anatomy. In pituitary surgery, this anatomical recognition facilitates the safe opening and entering of the sella, and enables maximal safe tumour resection, without collateral damage to critical neurovascular structures. However, this anatomical recognition is often challenging - reflected in the use of numerous adjuncts in modern practice, such as neuronavigation and micro-Doppler. A real-time, AI-driven, vision-based augmented reality display of anatomical structures may be a useful tool, used in synergy with existing adjuncts, to improve surgical anatomy recognition and surgical safety^5^. This could serve as a decision support for surgeons intra-operatively, toggling on to display anatomical predictions when required or as a warning system, and potentially reducing collateral damage (e.g. carotid injury) during tumour access and resection^5^.

The maximal benefit of AI assistance was realised by non-expert groups, with the benefit inversely proportional to the level of expertise, for the recognition of the centre and the total area of the sella. Medical students both utilised and benefitted from the AI assistance the most, followed by junior trainees and senior trainees, with their AI-assisted performance equivalent to that of an expert. This highlights the potential for such a technology in this context for training. The knowledge and recognition of anatomical structures is a core tenet of surgical training, but the majority of resources focus on selected and curated dissections and diagrams for education, with a growing use of AI to automatically label anatomical structures to augment educational yield from such materials^12^. Here, we demonstrate the use of AI to display anatomical structures on contemporary surgical images, with day-to-day challenges to anatomical recognition (e.g. bleeding, blurring, blocking by instruments). Offline, this could be used to generate indexed images of real-world surgical anatomy utilised by trainees to improve their anatomical recognition skills before an operation^5,13^. Similarly, this could be incorporated into educational procedure-specific assessments^13,14^. Moreover, with further refinements of the AI model’s performance, and through combination with augmented reality technology, this anatomical recognition overlay could be displayed to trainees in real-time intra-operatively to supplement their learning in practice^5,13,15^.

Furthermore, expert neurosurgeons both used AI assistance the least, improved the least when they used AI assistance and were least likely to reduce performance post AI assistance. For detection of the centre of the sella (i.e. safe entry zone), experts had a 100% recognition rate pre-AI assistance. However, when recognising the wider sella area, including the boundary and interface with surrounding structures (arguably a more difficult task), experts achieved a 73.4 DICE score, which improved by a marginal but statistically significant amount to 74.5 (*p* = 0.032). This may be because their baseline performance without AI was already high; therefore, any benefit attained was marginal. Alternatively, this may suggest experts did not trust the AI assistance as much as less experienced clinicians – either disregarding the AI recommendations (in 61% of images) or making minimal changes to their annotations when AI assistance was used. Determining the exact reasons for this was beyond the scope of this particular study; however, incorporation of human factors analysis (particularly trust and usability) will be crucial in the ongoing development of this technology (for example, as an intra-operative decision support tool), even at the lowest levels of AI autonomy^13,16,17^.

In the wider literature, surgical anatomy recognition is a developing space within the growing field of surgical video computer vision, building on the foundations laid by diagnostic image (e.g. radiological) analysis^18–22^. The majority of AI models use supervised deep learning based methods, being largely applied to surgeries during which the majority of the procedure is performed using endoscopes or microscopes (to allow video recording)^18–20^. The rapid expansion of this technology, particularly in laparoscopic surgery, has been facilitated by numerous public video datasets, open-source algorithms and co-ordinated community challenges^22–29^. In laparoscopic cholecystectomy, some AI models can recognise anatomical structures (e.g. common bile duct) and safe surgical zones more accurately and earlier in the operation than expert surgeons^30–35^. The wider laparoscopic surgery field has seen similar advancements in AI models for anatomy recognition in colorectal, urological and gynaecological applications^29,36–38^. Similarly, there are numerous examples of AI-driven anatomy analysis in endoluminal endoscopic procedures, for example, ampulla recognition in endoscopic retrograde cholangiopancreatography, and real-time vocal cord recognition in laryngoscopy and bronchoscopy^39,40^. Another notable example is polyp detection in colonoscopy, where accurate recognition was achieved for a range of case difficulties, outperforming experts, and subsequently translated into clinical practice as a decision support tool^28,41^. Within pituitary surgery, computer vision work has focussed on the recognition of surgical steps and phases^2,5,10,42,43^, with a single study exploring nasal anatomy recognition and another exploring tumour recognition^11,44^. In the future, many of these applications will integrate together (e.g. improved anatomy recognition will likely improve step recognition, and vice versa), and will interface with other technologies (e.g. augmented reality), for a variety of potential clinical applications^5,20,22^.

This pre-clinical comparative study has numerous strengths. Firstly, it is the first comparative study of an AI-driven decision support tool for skull base anatomy recognition to our knowledge. Additionally, we used a cross-over design to match baseline characteristics in the study groups, and attained a diverse sample of clinicians with varying expertise levels. There are, however, numerous limitations of this work. Our assessment was limited to six images for each of the 24 participants within an academic teaching hospital environment, and future studies should include more participants, across multiple centres, with larger assessments where possible. Furthermore, all sella predictions are currently offline, on still images, and future iterations with larger datasets and refined AI models will focus on displaying this AI recognition on real-time video, with a user interface tailored to the needs of surgeons, and metrics encompassing safety, effectiveness and efficiency.

In conclusion, in this pre-clinical comparative study, we have demonstrated the utility of AI assistance for students, trainees and experts in skull base anatomy recognition in endoscopic pituitary surgery. The less experienced the user, the more benefit was gained from AI assistance – both in terms of improving performance and safety. With AI assistance, trainees were able to achieve expert-level anatomy recognition proficiency. This technology, therefore, has the potential for use in augmenting surgical education, both for offline review and for real-time learning in practice. Further work is needed to improve real-time model performance, the user interface of displays, and integration with other anatomy recognition adjuncts, for use as an eventual intra-operative decision support tool.

## Methods

### Study design

A pre-clinical (IDEAL Stage 0) comparative study of clinician performance in sellar anatomy recognition, with and without AI assistance, was adopted—guided by DECIDE-AI and IDEAL reporting guidelines^16,45–47^. The study was based at a tertiary neurosurgical centre, the National Hospital for Neurology & Neurosurgery London, which acts as a regional referral centre for pituitary tumours and carries out approximately 150-200 pituitary operations each year. Ethical approval was granted via the Frenchay regional ethics committee (IRAS 271696), and informed written patient consent was obtained for the data used for AI model development.

### AI Model and Dataset Background

This study applies an artificial neural network previously developed by our team - capable of accurate recognition of the sella in endoscopic pituitary adenoma surgery^8,9^. This model was trained using an image dataset from 64 anonymized videos of endoscopic pituitary adenoma surgery. Ten images corresponding to the ten seconds of the sellar phase immediately preceding sellotomy were extracted from each video, resulting in 640 images in total^2,42^. Ground truth annotation of these images for the sella was performed with polygon labelling using the Touch Surgery^TM^ Ecosystem (Medtronic, Dublin, Ireland) via a multi-round expert consensus process. The sella was identified from endoscopic images by its bony protrusion into the sphenoid sinus, with its boundaries defined by peripheral contour changes (i.e. flattening of the prominence) and/or the beginning of an adjacent structure (e.g. carotid arteries)^4,48–50^. Firstly, each image was segmented in duplicate by trainee neurosurgeons [DZK, JGH, SW], with any differences settled through discussion. These segmentations were reviewed and adjusted by two consultant neurosurgeons [HJM, AB] independently, who had performed the source operations and had access to the full operative video of each image set to improve the contextualisation of images and support more accurate ground truth annotations. Interrater agreement for sella ground truth annotations was calculated using DICE scores (a measure of overlap between annotation masks) for 20 randomly selected images (from 20 cases). Interrater agreement was worse between trainees (DICE 76.4), best between consultants (DICE 85.2), and intermediate between trainees vs. consultants (DICE 80.2). This supported the need for multi-round, multi-expert verified ground truth labelling for model training.

### Comparative evaluation of human-only vs AI-augmented anatomy recognition

Six medical students, six junior neurosurgical trainees (in the first three years of training, without formal subspecialty experience in pituitary surgery), six intermediate trainees (over three years of training but without formal subspecialty experience in pituitary surgery) and six experts (consultants or senior fellows with subspecialty training in pituitary surgery) were recruited from our centre. Six video cases were selected randomly from the hold-out dataset, and one frame was selected per video from the available video images. Visualisation of all of the sella was not possible in all images due to obscuring blood, mucosa, bony septations and instruments, pathological distortions or anatomical variations^8,51^. Therefore, the selection of each frame was on the basis of its clarity, i.e. minimal blurring and obscuration (from blood, mucous, etc.). This six image dataset was presented in a random order to each participant.

**Fig. 2:**
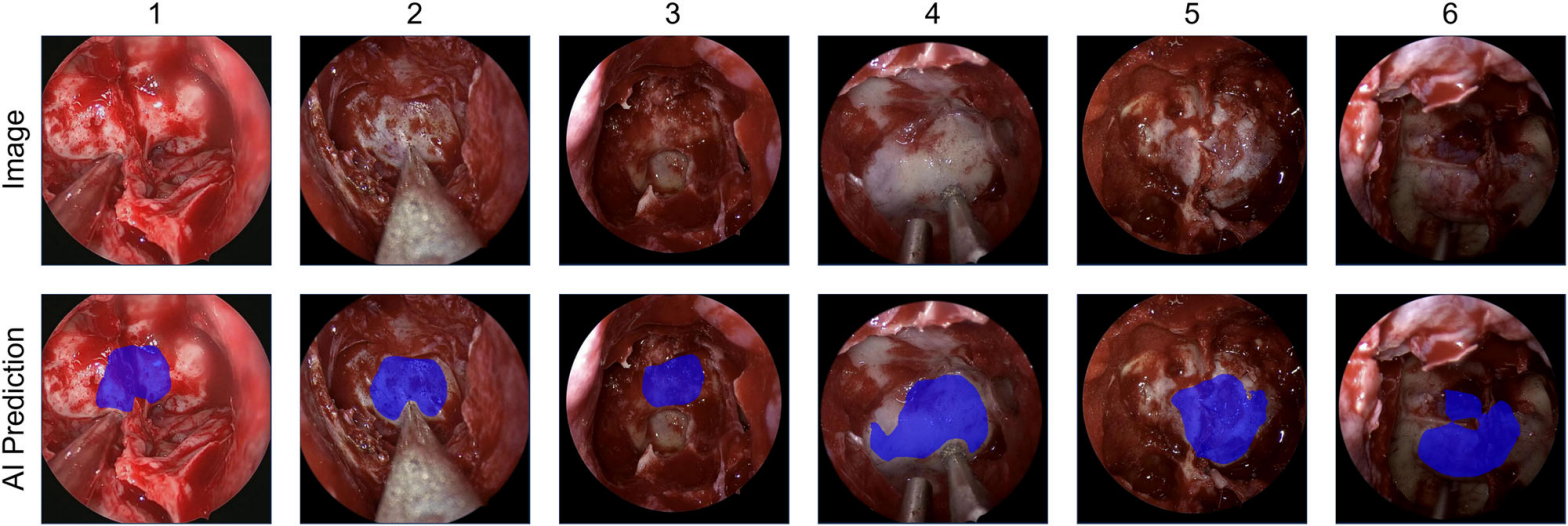
The six images used in this study in the order presented to the human participants (1–6). The top “Image” row shows the image without any AI assistance – this was presented to the participants and they added polygon labelling of anatomical structures. The bottom “AI Prediction” row shows the image with the AI predicted sella segmentation, which was presented to the participants as an overlay onto their anatomical labelling.

In round 1 (human only), each of the 24 human participants was asked to draw the outline of the sella on each of the six images without any adjuncts using the Touch Surgery^TM^ Ecosystem polygon annotation platform. If the sella was partly occluded, participants were instructed to draw around any obscuration. This was immediately followed by round 2 (AI-assisted), where the output of the AI model (a predicted sella outline) was overlayed onto their existing annotation for each image, and participants were invited to keep their existing annotations or alter their annotations based on AI recognition (Fig. 2).

Comparative analysis was performed between the human-only vs human + AI-assistance sella annotations, judging them against the aforementioned multi-round expert consensus labelling (best available ground truth). The mean DICE score was calculated for each subgroup (medical students, junior trainees, intermediate trainees and experts), with and without AI assistance. The Wilcoxon signed rank test was used to compare DICE scores per group and sub-group, maintaining the pairing for each participant-image combination. The number of participants with sella annotations encompassing the centroid of the ground truth label (i.e. including an approximate of the middle of the sella) across the six images was calculated for each subgroup, with and without AI assistance – compared using McNemar’s test. Statistical analysis was conducted using Python 3.8^52^ and R statistical programming^53^.

Additionally, the incidence of changes post AI assistance was explored descriptively, as well as the effect of these changes on DICE scores for sella recognition. Finally, error analysis was performed by calculating false positive and false negative predictions, both pre and post AI assistance. These are a focused analysis of areas of non-overlap, as opposed to the DICE score, which is a metric of overlap. False positive prediction was defined as the area labelled as sella by participants, which did not overlap with the ground truth sella label. False negative prediction was defined as the area of the ground truth sella label which did not overlap with the participant sella label. Both were calculated as a percentage of the total combined area of the overlap between the ground truth and participant labels.

## Data Availability

The data that support the findings of this study are available from the corresponding author upon request.

## Code availability

A 50-5-9 training-validation-test videos split was employed, and a Jaccard loss function and Adam optimiser was employed, with a batch size of 5 and run for 20 epochs^8^. The epoch with the highest mean Jaccard score of the sella segmentation mask on the validation dataset was kept as the final model^8^. The hold-out test dataset consisted of the first image of each of the 9 unseen videos (9 images), with a DICE score of 79.1^8^. The code is written in Python 3.8 using PyTorch 1.8.1, run on a single NVIDIA Tesla V100 Tensor Core 32-GB GPU using CUDA 11.2, and is available at https://github.com/dreets/pitnet-anat-public^8^.
